# Local ocular safety of the subconjunctival injection of cetuximab in rabbits

**DOI:** 10.1186/s12886-023-02893-6

**Published:** 2023-04-13

**Authors:** Hossein Aghaei, Ahmad Kheirkhah, Ali Mohammad Alizadeh, Acieh Es’haghi, Roshanak Aliakbar-Navahi, Zahra Keikha, Samira Chaibakhsh

**Affiliations:** 1 Eye Research Center, The Five Senses Health Institute, Rassoul Akram Hospital, Iran University of Medical Sciences, Tehran, Iran; 2 Department of Ophthalmology, Long School of Medicine, UT Health San Antonio, San Antonio, TX USA; 3 Cancer Research Center, Tehran University of Medical Sciences, Tehran, Iran; 4 Department of Ophthalmology, School of Medicine, Iran University of Medical Sciences, Tehran, Iran

**Keywords:** Ocular surface squamous neoplasia, Intraepithelial neoplasia, Cetuximab

## Abstract

**Background:**

To evaluate the safety of different doses of subconjunctival cetuximab in rabbits.

**Methods:**

After general anesthesia rabbits received a subconjunctival injection of 2.5 mg in 0.5 ml, 5 mg in 1 ml, and 10 mg in 2 ml of cetuximab in their right eyes (two rabbits in each group). A similar volume of normal saline solution was injected subconjunctivally in the left eyes. The histopathologic changes were evaluated after enucleation with the aid of H&E staining.

**Results:**

No significant difference were observed between the treated and control eyes in terms of conjunctival inflammation, goblet cell density, or limbal blood vessel density for all administered doses of cetuximab.

**Conclusion:**

Subconjunctival injection of cetuximab with the administrated doses in rabbit eyes are safe.

## Background

OSSN (ocular surface squamous neoplasia) is the most prevalent ocular surface tumor and the third most frequent ocular malignancy in adult population [1]. Currently, the term OSSN is widely used to include a spectrum of lesions ranging from CIN (intraepithelial neoplasia) to invasive SCC (squamous cell carcinoma) [2, 3]. Exposure to ultraviolet radiation, HPV (human papilloma virus) infection, immunosuppression, HIV (human immunodeficiency virus) infection and advanced age are considered as the main risk factors for occurrence and progression of OSSN [4].

Surgical excision with cryotherapy is considered to be the conventional management for OSSN [5]. However, this invasive approach is associated with potential risk of damaging limbal stem cells [6–8]. Recent advances in the chemotherapeutic treatments have enabled targeting of specific biological processes and pathways with the aid of agents, such as topical mitomycin C, topical 5-fluorouracil, and perilesional and topical interferon α-2b, that can be used either alone or as adjuncts to surgical excision of OSSN lesions [9–11]. However, application of such agents are associated with side effects such as punctate epithelial keratopathy, filamentary keratitis, corneal ulcers, limbal stem cell deficiency, symblepharon, and allergic reactions that emphasizes the importance of looking for newer therapeutics agents with more favorable and acceptable safety profile [11, 12].

Expression of EGFR (epidermal growth factor receptor) is upregulated in human epithelial malignancies, including the majority of SCCHN (squamous cell carcinoma of the head and neck) tumors (80–100%) [13]. EGFR has also been observed in tumor cell nuclei in OSSN lesions, showing the nuclear translocation and activation status of this receptor. Minimal or absence of EGFR reactivity has been documented in nearby normal conjunctival tissue [14]. Furthermore, it has been shown that expression of EGFR signaling pathway is associated with development of conjunctival squamous carcinoma [14, 15].Cetuximab is a chimeric monoclonal EGFR human/murine antibody which contains IgG1 constant region. As the affinity of cetuximab for EGFR is nearly 5 to 10 times of the endogenous ligands, it can block endogenous ligand binding, which reduces EGFR-mediated signaling and leads to growth inhibition of tumor cells [13, 15]. In fact, it has been shown that in vitro exposure of SCC cell lines to cetuximab prevents the proliferation of tumoral cells [15].

Here in this study, the safety of different doses of subconjunctival injection of cetuximab in rabbit eyes was evaluated to provide a foundation for further investigations regarding its efficacy as an alternative biological agent for the treatment of OSSN.

## Methods

This experimental study adhered to the Statement for the Use of Animals in Ophthalmic and Visual Research tenets [16]. The rabbits were obtained from Production and Research Complex, Pasteur institute of Iran. This study was performed in accordance with the Declaration of Helsinki and all experiments were performed in accordance with the Institutional Animal Ethics Committee of Iran university of medical sciences which governed the use of experimental animals.

Six healthy white New Zealand male rabbits, weighing 1.5–2 kg, were used for the study. Each rabbit underwent a complete systemic evaluation by a veterinarian. Moreover, an ophthalmologist assessed their eyes health status by a portable slit lamp. The animals were kept in a well-ventilated environment with a temperature of 25 ± 2° C and the humidity of 70% ± 5% during the study.

For subconjunctival injection of cetuximab, general anesthesia was performed for all the rabbits by intravenous 37.5 mg/kg ketamine hydrochloride (Rotexmedica, Tritau, Germany) and 5 mg/kg xylazine (Xylazine,Alfasan International, Netherlands). Cetuximab (Erbitux®, Merck,USA) and normal saline solution were prepared just prior to administration. The rabbits received a subconjunctival injection of 2.5 mg in 0.5 ml, 5 mg in 1 ml, and 10 mg in 2 ml of cetuximab in their right eyes (two rabbits in each group). A similar volume of normal saline solution was injected subconjunctivally in their left eyes. A 30-gauge needle was used for subconjunctival injections and all injections were performed at 2 mm behind the superotemporal limbus. No eye drops were given after subconjunctival injection.

Rabbits were examined using a portable slit lamp under general anesthesia on day 2, day 3, week 1, and month 1after the injection. Complete anterior segment evaluation was performed for the presence of any conjunctival reaction including inflammation or melting, corneal infiltration or melting, or anterior chamber inflammatory reaction. Furthermore, corneal fluorescein staining was performed to assess any corneal epitheliopathy or epithelial defect. At 1 month after the subconjunctival injection, the rabbits were euthanized with an intraperitoneal administration of a lethal dose (100 mg/kg) of thiopental (Panpharma, France). Enucleation of all eyes was performed by careful preservation of the conjunctiva. The conjunctiva were then used for histopathologic studies.

### Histological study

For histopathologic evaluation, enucleated globes were fixed in 10% formalin solution and bisected into two halves at the injection site, and then serial sections with 5-μm thickness with 25 μm intervals were prepared from the paraffin-embedded blocks. The pathologist performed histopathologic evaluation was blinded to the identity of any of the specimens represented on the slide with respect to group assignment.

H&E (hematoxylin and eosin) staining was used to evaluate the histopathologic changes in the conjunctiva and adjacent corneal and underlying scleral tissues regarding the presence of any inflammation, granuloma, stromal edema, tissue necrosis, and scar formation as well as evaluating the limbal blood vessels density. The integrity of epithelial basement membranes and number of goblet cells were also evaluated by PAS ( Periodic Acid Schiff) staining.

The inflammatory response and the number of goblet cells and blood vessels were assessed semi-quantitatively, using the following grading systems; The inflammation was classified as grade 1, mean inflammatory cells infiltration less than 10 per three HPFs (high power field); grade 2, mean inflammatory cells infiltration between 10 and 20 per three HPFs; and grade 3, mean inflammatory cells infiltration more than 20 per three HPFs. Goblet cell density was identified as grade 0 if no goblet cells were observed in three HPFs near the limbus, grade 1 if the mean number of goblet cells was less than 3 per three HPFs and as grade 2 if the mean number of goblet cells was greater than 4 per three HPFs. Limbal blood vessels density was calculated by taking the average of the number of thin wall vessels seen in three HPFs and identified as grade 1 with a mean blood vessel count/3 HPF less than five and grade 2 with a mean blood vessel count/3 HPF greater than six.

### Statistical analysis

All analysis were performed by SPSS version 24 and McNemar's test was performed to compare the control and treated eyes,. *P* values of < 0.05 were considered as statistically significant.

## Results

In follow-up visits, aside from trace subconjunctival hemorrhage and mild chemosis in both eyes of one rabbit that had received 10 mg/2 ml cetuximab, no other adverse events were observed. Chemosis and subconjunctival hemorrhage disappeared on day 3 and week 1 after the injection, respectively in this rabbit.

In histopathologic evaluation, the conjunctival tissue at the site of injection showed intact non-keratinized squamous epithelium and intact basement membrane in all treated groups. There was no significant difference between the treated eyes and the control eyes regarding conjunctival inflammation, goblet cell density, or limbal blood vessel density for all studied doses of cetuximab (Table 1).

**Table 1:** Statistical comparison between the control and treatment groups with different dosages of cetuximab injection regarding the histopathological parameters

**Histopathology Evaluation Items**	**Histological Grades**	**Intervention (%)**	**Placebo (%)**	***P*-value**
**Inflammation**	**Total (*N*=6)**			>0.999
	Grade1	5(83)	4(67)	
	Grade2	1(16.7)	2(33)	
	**2.5 mg (*N*=2)**			NA
	Grade1	2(100)	2(100)	
	Grade2	0	0	
	**5** **mg** **(*N*=2)**			>0.999
	Grade1	1(50)	1(50)	
	Grade2	1(50)	1(50)	
	**10 mg** **(*N*=2)**			NA
	Grade1	2(100)	1(50)	
	Grade2	0	1(50)	
**Goblet Cell**	**Total(*N*=6)**			>0.999
	Grade1	3(50)	3(50)	
	Grade2	3(50)	3(50)	
	**2.5 mg** **(*N*=2)**			>0.999
	Grade1	1(50)	1(50)	
	Grade2	1(50)	1(50)	
	**5 mg** **(*N*=2)**			NA
	Grade1	0	1(50)	
	Grade2	2(100)	1(50)	
	**10 mg** **(*N*=2)**			NA
	Grade1	2(100)	1(50)	
	Grade2	0	1(50)	
	**Total (*N*=6)**			NA
	Grade1	6(100)	6(100)	
	Grade2	0	0	
	**2.5 mg** **(*N*=2)**			NA
	Grade1	2(100)	2(100)	
	Grade2	0	0	
	**5 mg** **(*N*=2)**			NA
	Grade1	2(100)	2(100)	
	Grade2	0	0	
	**10 mg** **(*N*=2)**			NA
	Grade1	2(100)	2(100)	
	Grade2	0	0	

NA Not Applicable/Because of the small sample size, exact tests were utilized and existing zero cells (frequency of the samples of a grade in each group) made the inferences difficult and *p*-values could not be computed with the tests**; **mg**:** milligram

**Fig. 1:**
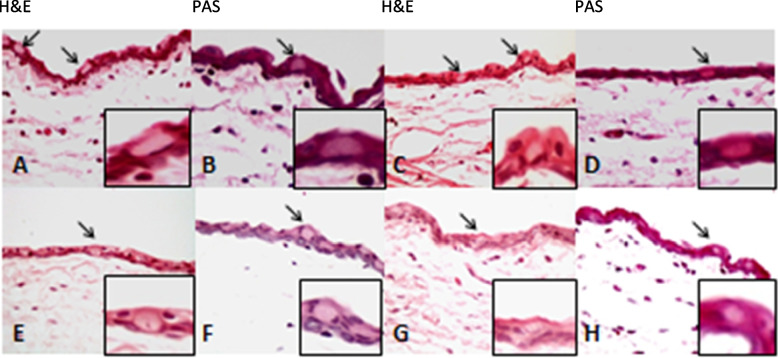
Histopathology images of the conjunctiva with H&E and PAS staining (X400) in treated group with 2.5 mg Cetuximab (**A-B, E–F**) and control group (**C-D, G-H**). Note the intact epithelium and basement membrane with normal goblet cells distribution (arrows) and unremarkable underlying stroma with grade 1 to 2 of chronic inflammation, the same as the control group with a normal stromal aggregation of mononuclear inflammatory cells. H&E: hematoxylin and Eosin, PAS: periodic acid Schiff

**Fig. 2:**
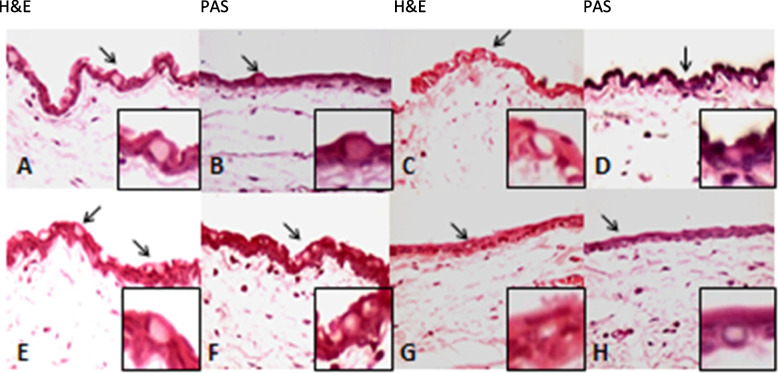
Histopathology images of the conjunctiva with H&E and PAS staining (X400) in treated group with 5 mg Cetuximab (**A-B, E–F**) and control group (**C-D, G-H**). Note the intact epithelium and basement membrane with normal goblet cells distribution (arrows) and unremarkable underlying stroma with grade 1 to 2 of chronic inflammation, the same as the control group with a normal stromal aggregation of mononuclear inflammatory cells. H&E: hematoxylin and eosin, PAS: periodic acid Schiff

**Fig. 3:**
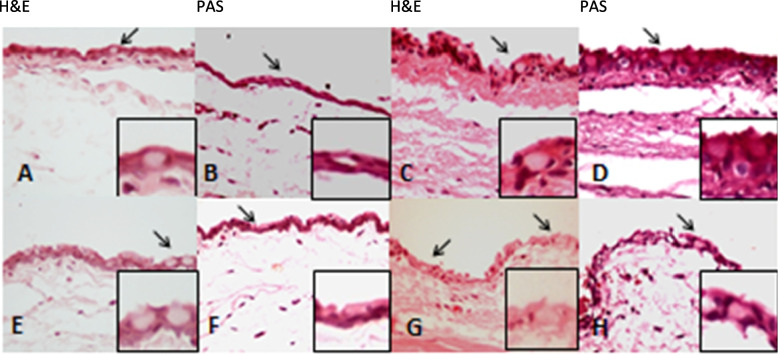
Histopathology images of the conjunctiva with H&E and PAS staining (X400) in treated group with 10 mg Cetuximab (**A-B, E–F**) and control group (**C-D, G-H**). Note the intact epithelium and basement membrane with normal goblet cells distribution (arrows) and unremarkable underlying stroma with grade 1 to 2 of chronic inflammation, the same as the control group with a normal stromal aggregation of mononuclear inflammatory cells. H&E: hematoxylin and eosin, PAS: periodic acid Schiff

**Fig. 4:**
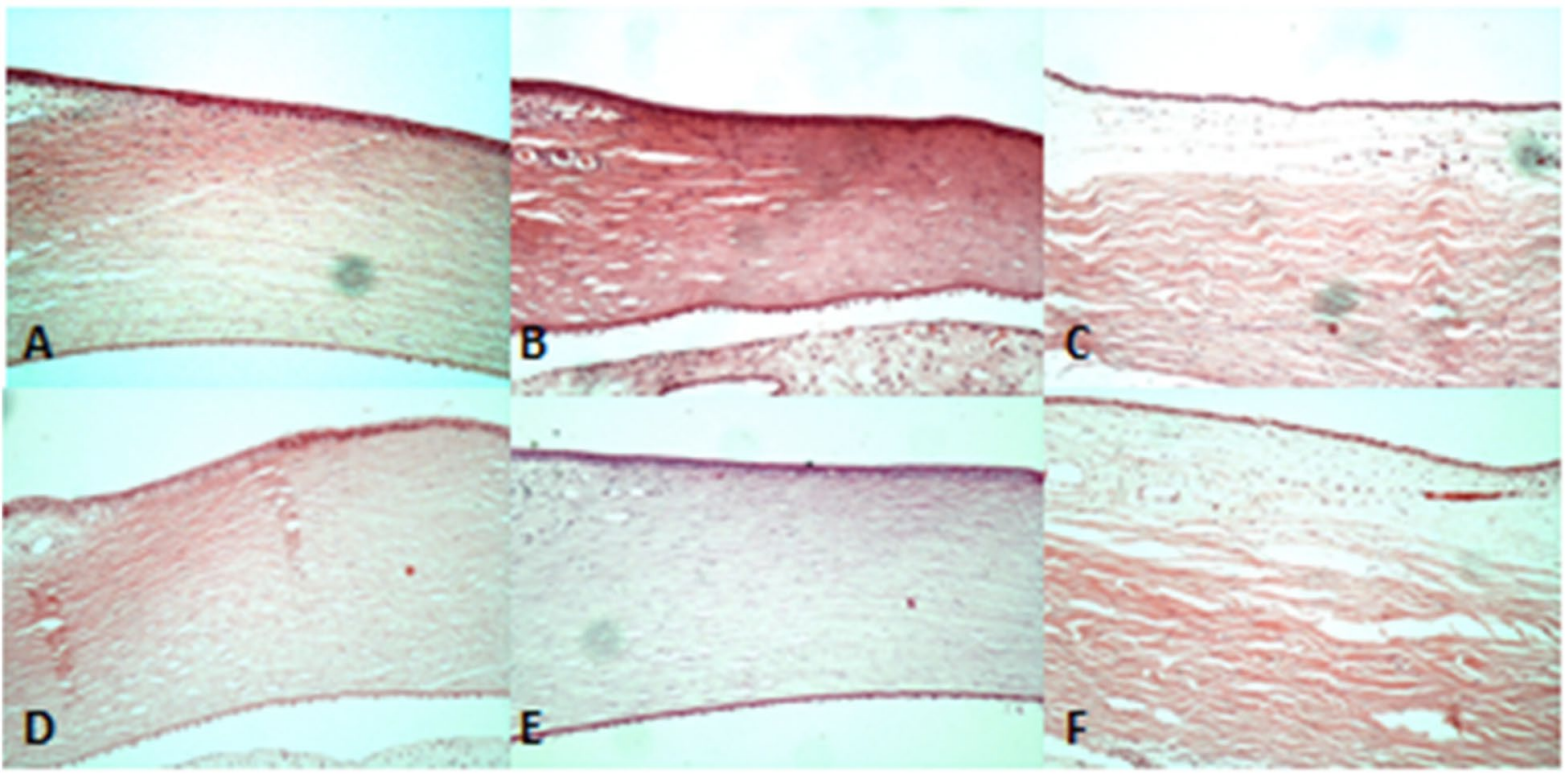
Histopathology images of treatment group (upper row) and control group (lower row) regarding the adjacent cornea and underlying sclera at the site of injection near the limbus: **A **&** D**: Cornea with intact squamous epithelium and stroma with normal distribution of keratocytes and absence of edema, inflammation and necrosis as well as well-preserved endothelial cells (H&E staining, × 100). **B **&** E**: PAS staining shows intact Descemet’s membrane (× 100). **C **&** F**: Scleral tissue under the conjunctiva and Tenon’s capsule with the normal collagen fibers shows absence of inflammation or necrosis (H&E staining, × 100)

Granulation formation, edema, and scar tissue were not observed in the conjunctival stroma of all treated groups (Figs. 1, 2, and  3). The adjacent cornea in the treated group had intact non-keratinized squamous epithelium with normal distribution of keratocytes in the stroma and absence of edema, inflammation and necrosis as well as well-preserved endothelial cells. Additionally, the underlying sclera of all treated groups did not show any significant histopathologic changes or thinning compared to the control group (Fig. 4).

## Discussion

Cetuximab is a chimeric and monoclonal antibody thatbinds human EGFR and inhibits its function. It also targets tumor cells with EGFR expression [17, 18]. Cetuximab has received the U.S.FDA (Food and Drug Administration) approval for the treatment of patients with loco-regional advanced SCCHN [19].

Ocular toxicity of systemic cetuximab is not common. Few reports stated cutaneous erythematous eruptions in the periorbital area, blepharitis, and conjunctivitis associated with systemic administration of the drug in patients with advanced colorectal carcinoma [20, 21]. There are limited data on toxicity of subconjunctival injection of cetuximab which can be a potential treatment for OSSN. Subconjunctival injection route is a good choice in patients who cannot use topical drugs for a long time and provides higher intra-lesion concentrations that may be associated with better drug efficiency [22]. Subconjunctival cetuximab has already been investigated in experimental model of corneal angiogenesis [23].

In this study, slit lamp biomicroscopy after subconjunctival injection of cetuximab showed only transient chemosis in a rabbit eye which was injected with 10 mg/2 ml of the drug. Such chemosis may be due to volume of the administrated drug. No other significant changes were noted in the examination of the other eyes during a follow-up of 1 month after the injection.

Also, histopathologic evaluation with H&E and PAS did not show any signs of toxicity in the conjunctival epithelium or stroma and the underlying cornea and sclera. There were no significant differences between the treated and control eyes in terms of conjunctival inflammation, goblet cell density, or limbal blood vessel density for all different doses of cetuximab. Tunik et al. [23] investigated the therapeutic effects of subconjunctival injection of cetuximab and/or bevacizumab on rats with induced corneal angiogenesis. The rats were divided into four groups. Group 1: 0.15 ml saline solution, Group 2: 1.25 mg bevacizumab, Group 3: 5 mg cetuximab, and Group 4: 1.25 mg bevacizumab plus 5 mg cetuximab. All eyes received the injections on days 1, 4, and 7 of the study. Findings of histopathology and immunohistochemistry demonstrated that the angiogenesis decreased significantly in both the bevacizumab and cetuximab groups. Less inhibition of angiogenesis was found in the bevacizumab plus cetuximab group. Thus, they suggested that treatment with cetuximab may be useful in management of corneal angiogenesis. However, they did not mention safety parameters.

Our study has some limitations. The main limitation is the small sample size in each group that restricts the interpretation of our findings. In addition, using just H&E and PAS staining without specific assays for cellular toxicity including molecular and immunohistochemical techniques may fail to detect some pathologic findings. However, our results can provide a starting point for the development of subconjuctival cetuximab-based future investigations.

## Conclusion

Our results showed that administrated doses of cetuximab in this study (2.5 mg/0.5 ml, 5 mg/1 ml, and 10 mg/2 ml) are possibly safe for subconjunctival injection in rabbit eyes. However, the efficacy study is needed to show the antitumor effects of these doses on OSSN. Further studies with larger sample size and long-term follow-up are necessary to confirm the safe dose and determine the antitumor properties.

## Data Availability

The datasets used and/or analyzed during the current study are available from the corresponding author on reasonable request.
